# Supplementary material for: Time-series transcriptome analysis identified differentially expressed genes in broiler chicken infected with mixed Eimeria species
Source: Front Genet. 2022 Aug 8;13:886781. doi: 10.3389/fgene.2022.886781 (PMC9393255; doi:10.3389/fgene.2022.886781)
Supplement: Supplementary file 2 [file DataSheet1.ZIP › 7dpi_GO.Gsea.1625106495923/GOBP_POSITIVE_REGULATION_OF_SMOOTH_MUSCLE_CELL_MIGRATION.html]

Details for gene set GOBP\_POSITIVE\_REGULATION\_OF\_SMOOTH\_MUSCLE\_CELL\_MIGRATION[GSEA]

|  || Dataset | TMM\_7dpi\_gct\_format\_7dpi\_gct\_format.Class\_4dpi.cls #PC\_versus\_NC.Class\_4dpi.cls #PC\_versus\_NC\_repos |
| Phenotype | Class\_4dpi.cls#PC\_versus\_NC\_repos |
| Upregulated in class | 0 |
| GeneSet | GOBP\_POSITIVE\_REGULATION\_OF\_SMOOTH\_MUSCLE\_CELL\_MIGRATION |
| Enrichment Score (ES) | -0.6041514 |
| Normalized Enrichment Score (NES) | -1.8458334 |
| Nominal p-value | 0.0 |
| FDR q-value | 0.09674232 |
| FWER p-Value | 0.7792 |
Table: GSEA Results Summary

  

Fig 1: Enrichment plot: GOBP\_POSITIVE\_REGULATION\_OF\_SMOOTH\_MUSCLE\_CELL\_MIGRATION      
 Profile of the Running ES Score & Positions of GeneSet Members on the Rank Ordered List

  

| SYMBOL | TITLE | RANK IN GENE LIST | RANK METRIC SCORE | RUNNING ES | CORE ENRICHMENT || 1 | S100A11 | na | 1500 | 0.381 | -0.1065 | No |
| 2 | BCL2 | na | 2059 | 0.261 | -0.1403 | No |
| 3 | ADAMTS1 | na | 2206 | 0.236 | -0.1409 | No |
| 4 | PDGFD | na | 3313 | 0.085 | -0.2291 | No |
| 5 | IGFBP5 | na | 3461 | 0.067 | -0.2381 | No |
| 6 | PDGFB | na | 4266 | -0.026 | -0.3039 | No |
| 7 | POSTN | na | 4322 | -0.032 | -0.3069 | No |
| 8 | LPAR1 | na | 4729 | -0.074 | -0.3372 | No |
| 9 | TERT | na | 5145 | -0.116 | -0.3661 | No |
| 10 | SRC | na | 6405 | -0.244 | -0.4593 | No |
| 11 | CRK | na | 6900 | -0.297 | -0.4859 | No |
| 12 | SSH1 | na | 7297 | -0.341 | -0.5023 | No |
| 13 | FGF9 | na | 7401 | -0.352 | -0.4936 | No |
| 14 | PDGFRB | na | 8726 | -0.517 | -0.5788 | Yes |
| 15 | PAK1 | na | 8943 | -0.541 | -0.5703 | Yes |
| 16 | LRP1 | na | 9005 | -0.550 | -0.5484 | Yes |
| 17 | NRP1 | na | 9452 | -0.624 | -0.5550 | Yes |
| 18 | RPS6KB1 | na | 9556 | -0.639 | -0.5323 | Yes |
| 19 | NR4A3 | na | 10077 | -0.725 | -0.5401 | Yes |
| 20 | FOXO4 | na | 10103 | -0.729 | -0.5064 | Yes |
| 21 | DOCK7 | na | 10209 | -0.751 | -0.4784 | Yes |
| 22 | HAS2 | na | 10235 | -0.755 | -0.4434 | Yes |
| 23 | MDK | na | 10278 | -0.765 | -0.4093 | Yes |
| 24 | CCL5 | na | 10974 | -0.929 | -0.4218 | Yes |
| 25 | DOCK4 | na | 11087 | -0.960 | -0.3840 | Yes |
| 26 | ITGA2 | na | 11325 | -1.048 | -0.3524 | Yes |
| 27 | SEMA6D | na | 11527 | -1.127 | -0.3139 | Yes |
| 28 | DOCK5 | na | 11619 | -1.184 | -0.2634 | Yes |
| 29 | VTN | na | 11634 | -1.193 | -0.2060 | Yes |
| 30 | IGF1 | na | 11859 | -1.426 | -0.1548 | Yes |
| 31 | HDAC4 | na | 11924 | -1.578 | -0.0827 | Yes |
| 32 | NOX4 | na | 11976 | -1.831 | 0.0028 | Yes |
Table: GSEA details [plain text format]

  

Fig 2: GOBP\_POSITIVE\_REGULATION\_OF\_SMOOTH\_MUSCLE\_CELL\_MIGRATION      
 Blue-Pink O' Gram in the Space of the Analyzed GeneSet

  

Fig 3: GOBP\_POSITIVE\_REGULATION\_OF\_SMOOTH\_MUSCLE\_CELL\_MIGRATION: Random ES distribution      
 Gene set null distribution of ES for **GOBP\_POSITIVE\_REGULATION\_OF\_SMOOTH\_MUSCLE\_CELL\_MIGRATION**
